# Supplementary material for: Single-cell tumor-immune microenvironment of BRCA1/2 mutated high-grade serous ovarian cancer
Source: Nat Commun. 2022 Feb 11;13:835. doi: 10.1038/s41467-022-28389-3 (PMC8837628; doi:10.1038/s41467-022-28389-3)
Supplement: Supplementary file 1 — Supplementary Information [file 41467_2022_28389_MOESM1_ESM.pdf]

Single-cell tumor-immune microenvironment of BRCA1/2 mutated high-grade serous ovarian cancer

Supplementary Information

Contents:

Supplementary Figure 1: Single-cell cell type annotations show distinct marker expression profiles and correlate with conventional pathological scoring

Supplementary Figure 2: Various immune cell subpopulations associate with PFI and immune diversity

Supplementary Figure 3: Tumor cell metaclusters show distinct marker expression profiles and associations to the HR-genotypes

Supplementary Figure 4: PCA plots show differential cell type co-occurrence patterns in patients with long and short PFI as well as in patients with different HR-genotype

Supplementary Figure 5: Ki67 expression in proliferating epithelial cells varies according to cellular neighborhoods

Supplementary Table 1. Antibodies used in t-Cycif protocol

Supplementary Table 2. Cox regression for immune cell subtypes

Supplementary Table 3. Cox regression for spatial neighborhoods and proliferation status

Supplementary Fig. 1

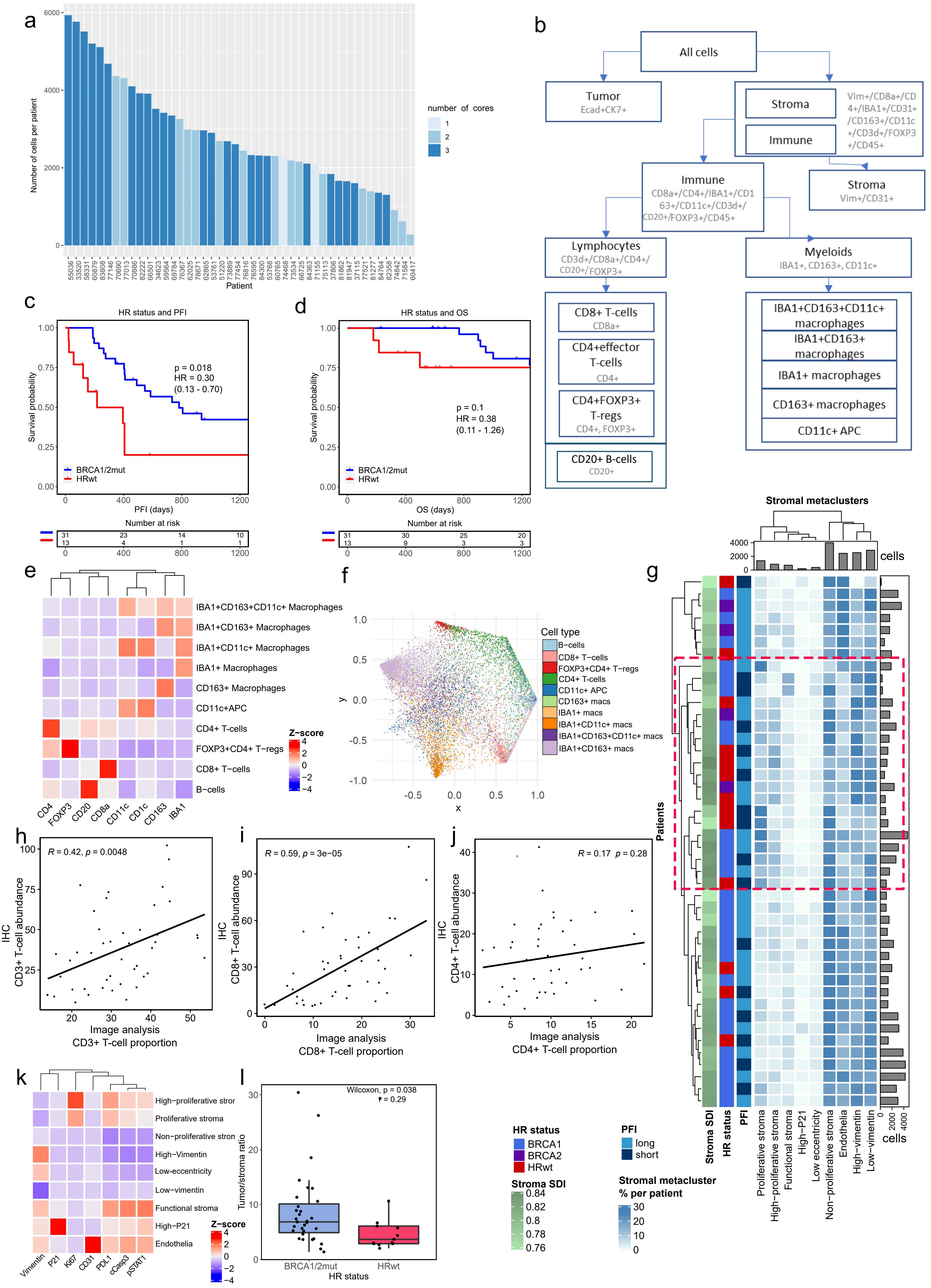

Supplementary Fig 1. Single-cell cell type annotations show distinct marker expression profiles and correlate with conventional pathological scoring

a Barplot of the number of TMA cores and single cells by patient. The color of the bars represents the number of cores per patient. **b** Using sequential annotation, we first identified tumor, immune and stromal cells, after which we annotated the immune subtypes. Markers used for annotation are shown in the flowchart. **c** Kaplan-Meier graph for PFI and **d** for OS grouped by the HR status. Number of patients at risk is shown at the bottom of each Kaplan-Meier graph. P-values were calculated by the log-rank test. **e** Heatmap of the Z-score of marker expressions across the annotated immune subtypes shows distinct expression profiles of the cell type markers. **f** Lineage trajectory plot separates cells of lymphoid and myeloid lineages from each other into opposite directions. **g** A hierarchical clustering heatmap of stromal metacluster proportions out of all stromal cells, annotated with stromal diversity (SDI), HR status and PFI. The barplot annotations for the columns and rows represent the total number of cells of each metacluster and the number of stromal cells in total per patient, respectively. **h** Linear correlation of imaging-based CD3+ lymphocyte annotations (CD8+T-cells, CD4+T-cells and FOXP3+CD4+T-regulatory cells) and pathological score of the count of lymphocytes from areas of high CD3+ lymphocyte infiltration from conventional immunohistochemistry (IHC). **i** Linear correlations of imaging-based CD8+T-cell annotations and **j** of CD4+T-cell annotations and pathological scores from conventional immunohistochemistry. The imaging-based annotations were calculated as a proportion out of immune cells. All counts from conventional immunohistochemistry were performed from selected fields enriched for CD3+cells. Spearman correlation coefficients and their p-values (no FDR adjustment) are shown. **k** A heatmap across the stromal metaclusters shows distinct expression profiles of the functional state markers. Hierarchical clustering was performed for the columns. **l** Boxplot showing an increased ratio of tumor to stromal cells in *BRCA1/2*mut (n=31) as compared to HRwt (n=13) tumors. The difference between the groups was calculated by two-tailed Wilcoxon rank-sum test. Boxplot visualizes sample medians, first to third quartiles and whiskers indicate values at 1.5 times the interquartile range**.** Individual dots represent values per tumor. Source data are provided with this paper.

Supplementary Fig. 2

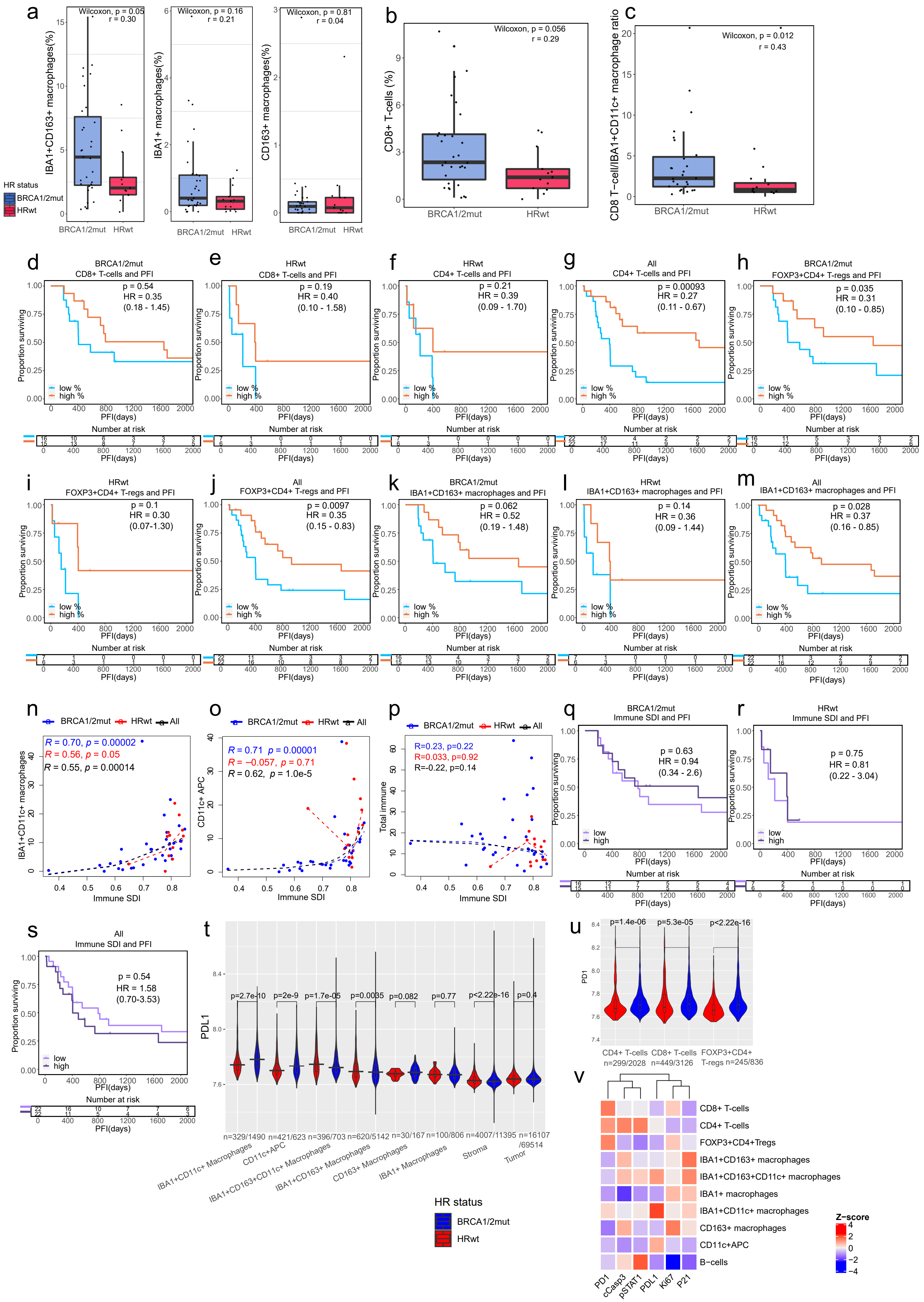

Supplementary Fig. 2 Various immune cell subpopulations associate with PFI and immune diversity

a Boxplots showing the proportions of IBA1+CD163+, IBA1+ and CD163+ macrophages and **b** CD8+T-cells as a proportion of all cells, and **c** boxplot showing the ratio of CD8+T-cells to IBA1+CD11c+ macrophages as a proportion of immune cells, stratified by HR status. The differences between the groups were calculated by two-sided Wilcoxon rank-sum test. Boxplots visualize sample medians, first to third quartile, and whiskers indicate values at 1.5 times the interquartile range**.** Individual dots represent values per tumor (n=31 *BRCA1/2*mut, n=13 HRwt). **d** Kaplan-Meier graphs for PFI for the proportion of CD8+T-cells out of all cells in patients with *BRCA1/2*mut tumors and **e** HRwt tumors. **f** Kaplan-Meier graphs for PFI for the proportion of CD4+T-cells in patients with HRwt tumors and **g** in all patients. **h** Kaplan-Meier graph for PFI for FOXP3+CD4+T-regulatory cells in patients with *BRCA1/2*mut and **i** HRwt tumors as well as **j** in all patients. **k** Kaplan-Meier graphs for PFI for IBA1+CD163+ macrophages in patients with *BRCA1/2*mut tumors, **l** HRwt tumors and **m** in all patients. **n** Scatter plots with lowess regression of immune diversity (SDI) and the proportion of CD11c+APCs and **o** the proportion of IBA1+CD11c+ macrophages out of immune cells and **p** total immune cells as a proportion of all cells, stratified by HR status. Black dashed line represents all tumors pooled. Spearman correlation coefficients and their p-values are shown. **q** Kaplan-Meier graphs for PFI for immune diversity in patients with *BRCA1/2*mut tumors, **r** HRwt tumors and **s** in all patients. The median was used as cutoff for the immune cell proportions and diversity. Number of patients at risk is shown at the bottom of each Kaplan-Meier graph. P-values were calculated using the log-rank test. **t** Violin plots showing the probability density of myeloid, tumor, and stromal cells and their PDL1 expression and **u** CD4+T-cells, CD8+T-cells, and FOXP3+CD4+T-regulatory cells and their PD1 expression, stratified by HR status. Boxplots show the sample medians and the first and third quartiles. P-values were calculated using two-tailed Wilcoxon rank-sum test and FDR corrected. **v** Heatmap of functional marker expressions (Z-score) across the immune subtypes. Hierarchical clustering was performed for the columns. Source data are provided with this paper.

Supplementary Fig. 3

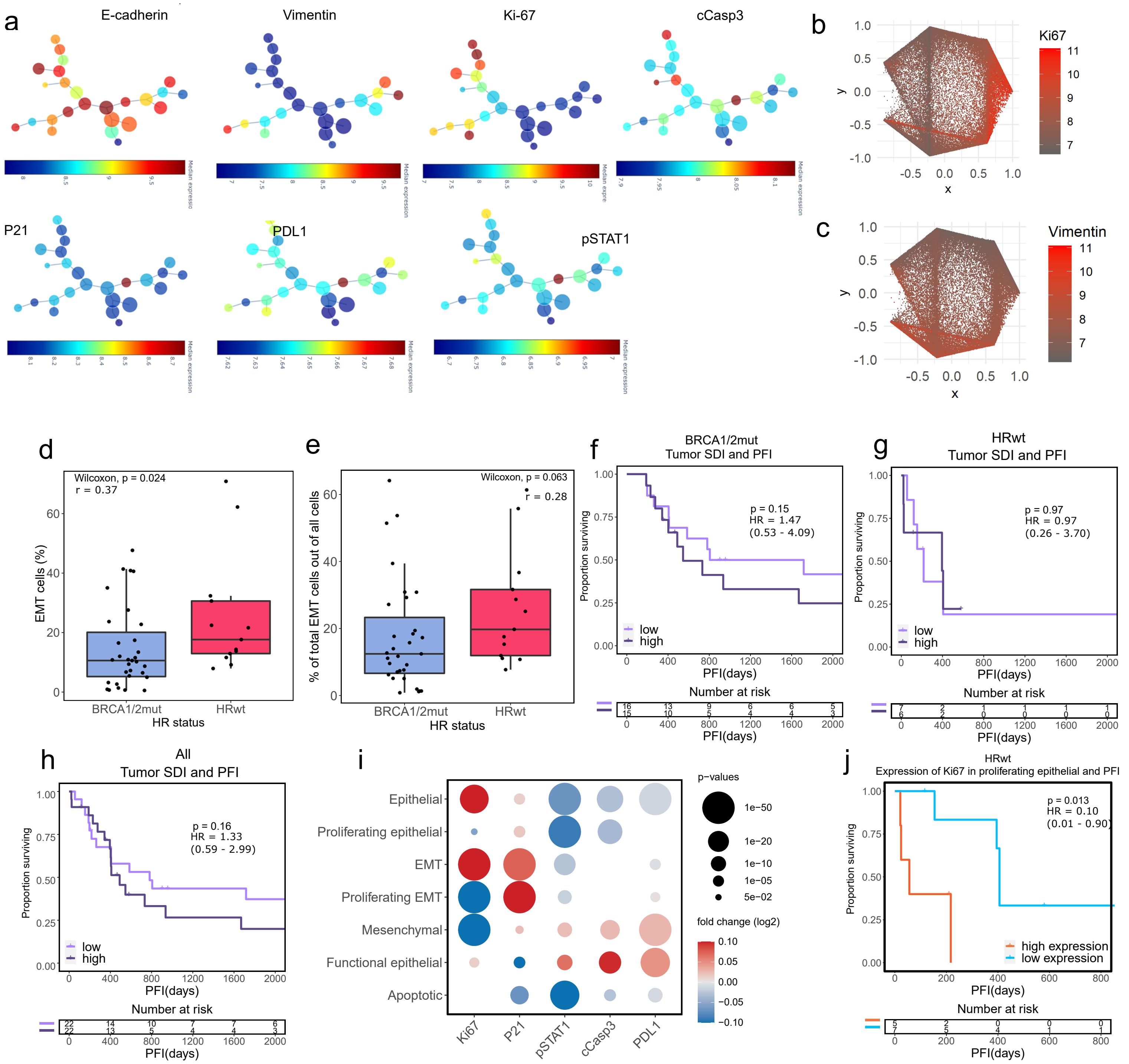

Supplementary Fig. 3 Tumor cell metaclusters show distinct marker expression profiles and associations to the HR-genotypes

a Minimum spanning trees of tumor cell clusters, showing differential median marker expression patterns in the tumor clusters. Lineage trajectory plot for tumor cell metaclusters colored by **b** Ki67 expression and **c** vimentin expression. **d** Boxplot showing the proportion of EMT cells as a proportion of all cells and **e** the total proportion of EMT cells and proliferating EMT cells as a proportion of all cells, stratified by HR status (n=31 *BRCA1/2*mut, n=13 HRwt). The difference between the groups was calculated by two-tailed Wilcoxon rank-sum test. The black horizontal lines represent the sample medians, the boxes extend from first to third quartile and whiskers indicate the values at 1.5 times the interquartile range**.** Individual dots represent values per each tumor. **f** Kaplan-Meier graphs show no association for PFI for tumor diversity index in patients with *BRCA1/2*mut (n=31) tumors, **g** in HRwt (n=13) tumors or **h** in all patients (n=44). Median values were used as a cut-off for high and low tumor diversity. **i** Dot plot of fold changes (log2) of functional marker expression between tumor metaclusters in *BRCA1/2*mut (n=31) and HRwt (n=13) tumors. The color of the dots represent the fold change and their size the significance of the p-value. Fold changes with FDR corrected p-values <0.05 are shown. **j** Kaplan-Meier graph showing low median Ki67 expression associates with an improved PFI in patients with HRwt tumors. Patients belonging to the top 1/3 percentile in all tumors in median Ki67 expression were annotated as high (n=5) and the rest as low (n=7). Number of patients at risk is shown at the bottom of each Kaplan-Meier graph. P-values were calculated using the log-rank test. Source data are provided with this paper.

Supplementary Fig. 4

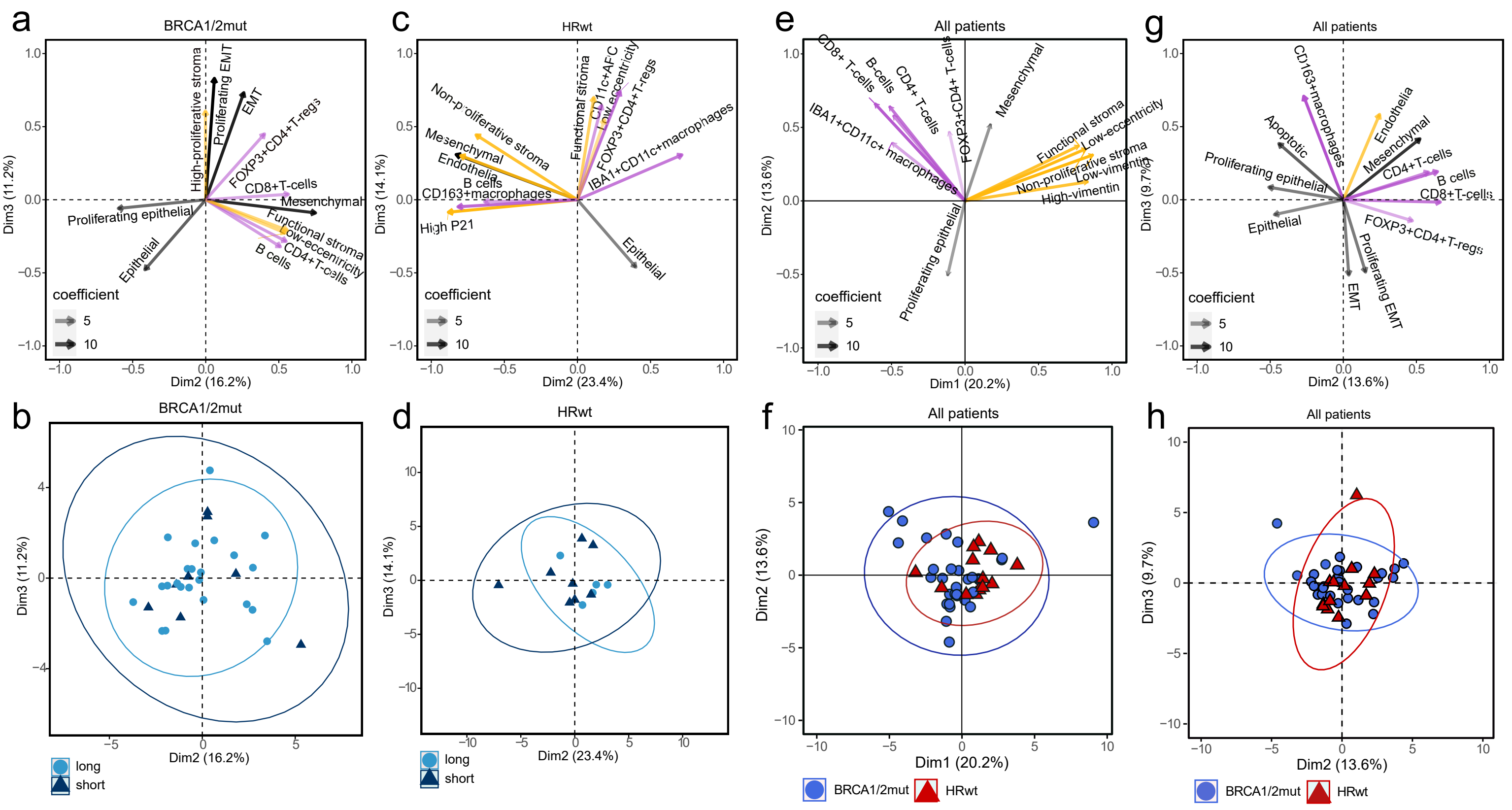

Supplementary Fig 4. PCA plots show differential cell type co-occurrence patterns in patients with long and short PFI as well as in patients with different HR-genotype

a Principal component feature projections for PC2 and PC3 for *BRCA1/2*mut tumors (n=31) and **b** plot of patients with *BRCA1/2*mut tumors projected onto the second and third principal component. **c** Principal component feature projections for PC2 and PC3 for HRwt tumors (n=13) and **d** plot of patients with HRwt tumors projected onto the second and third principal component. Patients are annotated as having a long or short PFI and colored by light blue or dark blue, respectively. Ellipsoids show the 95% confidence intervals for the PFI groups. 12 cell types with the highest contributions to PC2 and PC3 are shown for each plot. The more intense the color of the cell type, the higher its coefficient is. **e** Principal component feature projections for PC1 and PC2 for all tumors (n=44) and **f** plot of all patients projected onto first and second principal component. **g** Principal component feature projections for PC2 and PC3 for all tumors and **h** plot of all patients projected onto second and third principal component. Colors in principal component feature projections represent the global cell types (grey=tumor, yellow=stroma, purple=immune). Patients are annotated as having a *BRCA1/2*mut or HRwt tumor and colored by light blue or red, respectively. Ellipsoids show the 95% confidence intervals for the HR groups. 12 cell types with the highest contributions to PC1 and PC2, and PC2 and PC3 are shown for each plot. The more intense the color of the cell type, the higher its coefficient is. Source data are provided with this paper.

Supplementary Fig. 5

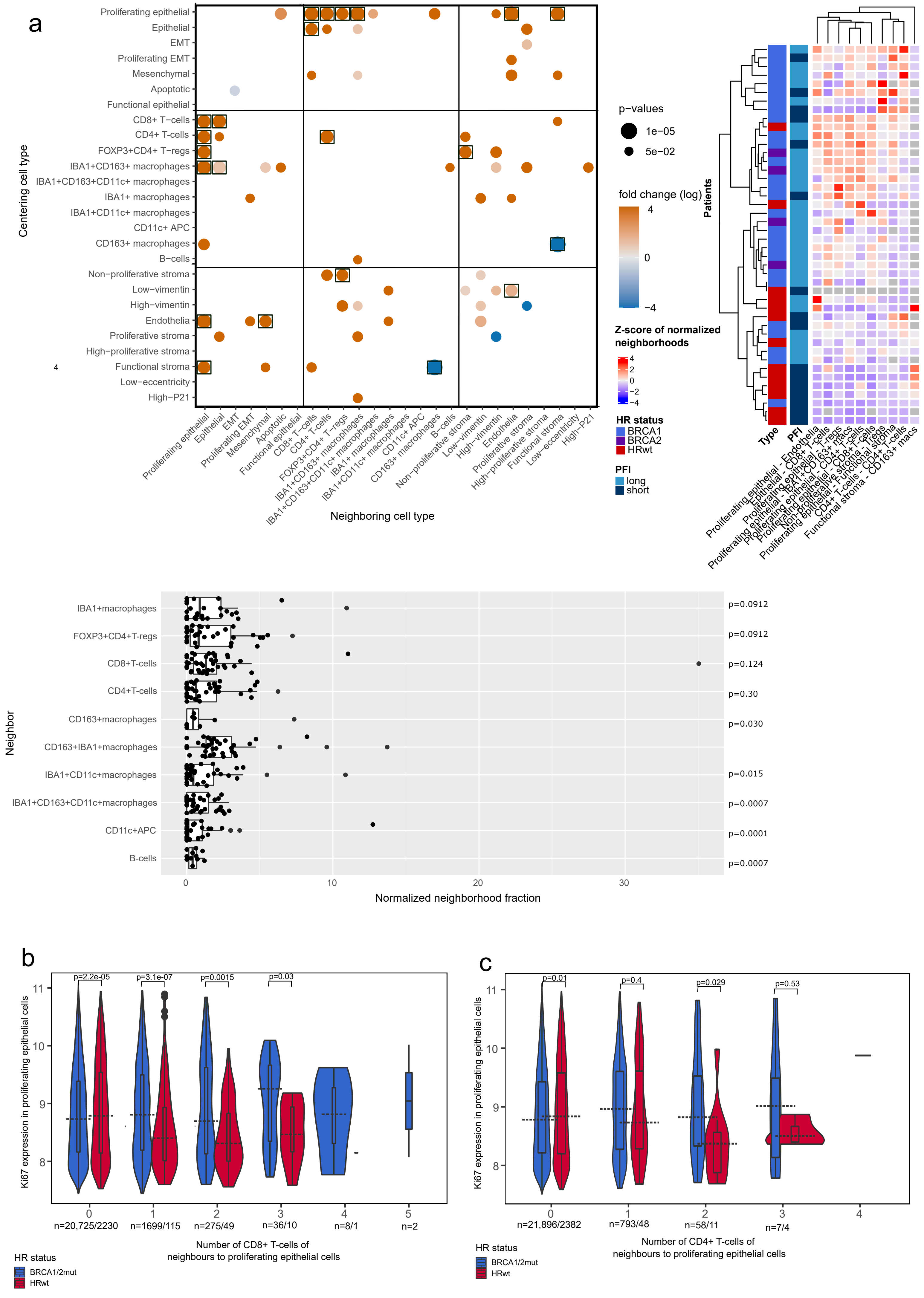

Supplementary Fig. 5 Ki67 expression in proliferating epithelial cells varies according to cellular neighborhoods

a Dot plot showing the fold change (log2) between *BRCA1/2*mut (n=31) and HRwt (n=13) tumors of the mean proportions of different cell types in the neighborhood (y-axis) of each centering cell type (x-axis). Orange colored dots represent more frequently occurring cellular neighborhoods in the *BRCA1/2*mut as compared to HRwt tumors, while blue dots represent more frequently occurring cellular neighborhoods in the HRwt as compared to *BRCA1/2*mut tumors. The size of the dot represents the significance of the p-value. P-values were calculated with two-tailed Wilcoxon rank-sum test. Only fold changes with a p-value of <0.05 are shown. Those p-values passing FDR < 0.1 are highlighted with a black square. Black lines separate tumor metaclusters, immune subtypes and stromal metaclusters from each other. Red dashed boxes highlight specific interactions. **b** A hierarchical clustering heatmap of bidirectional cell-cell spatial interactions presented in Fig.5a shows differential enrichment of spatial cell-cell interactions in the *BRCA1/2*mut and HRwt tumors. **c** Boxplot showing normalized neighborhood fractions of immune cell subtypes neighboring endothelial cells. Boxplots visualize the sample medians, first to third quartile and the values at 1.5 times the interquartile range. Individual dots represent values per tumor (n=44). FDR-corrected p-values for each comparison against IBA1+CD163+ macrophages are presented on the right side of the boxplot. P-values were calculated with two-tailed Wilcoxon rank-sum test. **d** Violin plots showing the probability density of proliferating epithelial cells and their Ki67 expression, stratified by HR status and the number of CD8+T-cells in the neighborhood and **e** showing the probability density of proliferating epithelial cells and their Ki67 expression, stratified by HR status and the number of CD4+T-cells in the neighborhood. Boxplots inside violin plots show the sample medians, the first and third quartiles, and whiskers show the 1.5x interquartile range. P-values were calculated with two-tailed Wilcoxon rank-sum test (no FDR correction). Source data are provided with this paper.

Supplementary Table 1. Antibodies used in t-Cycif protocol

Antibody	Fluorochrome	Company	Cat number	Clone	Concentration	Purpose
Rabbit 488	488	Thermo Fisher	A11034			Background
Rat 555	555	Thermo Fisher	A-21432			Background
Mouse 647	647	Thermo Fisher	A32728			Background
CD11c	Rabbit	CST	45581S		200	Dendritic cells
CD1c	Mouse	abcam	ab156708	GR222523-26	200	Dendritic cells
CD4	488	R&D	fab8165g		150	T-cells
CD3d	555	Abcam	ab208514		200	T-cells
CD20	647	eBioscience	50-0202-80		800	B-cells
CD163	488	Abcam	ab218293		500	M2 macrophages
CD8a	eFluor 660	eBioscience	50-0008-80		200	T-cells
cCasp3	488	CST	9969S	D175	100	Apoptosis
pSTAT1	555	CST	8183S		100	Interferon signalling
Ki67	555	eBioscience	41-5699-80		200	Proliferation
PD-L1	647	CST	CST 15005S		200	ICP
IBA1	488	Abcam	ab195031		800	Macrophages/myeloid cells
FOXP3	555	EbioSciences	41-477782		200	T-regulatory cells
PD1	647	Abcam	ab201825		200	ICP
E-cadherin	488	CST	3199		400	Tumor cells
Vimentin	555	CST	9855		100	Stroma
CD31	647	Abcam	218582		400	Stroma and endothelia
P21	488	CST	5487		200	Cell cycle
CK7	555	Abcam	ab209601		200	Tumor cells
CD45	647	Biolegend	304020		200	Immune cells

Supplementary Table 2. Cox regression for immune cell subtypes

**All patients**			
	**HR**	**95% CI**	**p-val**
CD4+ T-cells	0.34	0.14-0.85	0.02*
CD8+ T-cells	1.74	0.35-8.54	0.50
IBA1+CD163+ macrophages	0.60	0.26-1.39	0.23
FOXP3+CD4+ T-regs	0.92	0.40-2.11	0.84
HR status	3.11	1.29-7.46	0.01*
BRCA1/2**mut patients**			
	**HR**	**95% CI**	**p-val**
CD4+ T-cells	0.24	0.06-0.95	0.043*
FOXP3+CD4+ T-regs	0.59	0.17-2.08	0.41

Supplementary Table 3. Cox regression for spatial neighborhoods and proliferation status

BRCA1/2**mut, for PFI**			
	HR	95% CI	p-val
CD4+ T-cell neighbors	0.50	0.15-1.62	0.25
CD8+ T-cell neighbors	0.76	0.26-2.19	0.61
Ki67 in proliferating epithelial cells	0.15	0.02-1.22	0.08
BRCA1/2**mut, for OS**			
	HR	95% CI	p-val
CD4+ T-cell neighbors	0.24	0.05-1.18	0.08
CD8+ T-cell neighbors	0.96	0.25-3.75	0.96
Ki67 in proliferating epithelial cells	0.51	0.09-2.87	0.45
