# Supplementary material for: Single-cell tumor-immune microenvironment of BRCA1/2 mutated high-grade serous ovarian cancer
Source: Nat Commun. 2022 Feb 11;13:835. doi: 10.1038/s41467-022-28389-3 (PMC8837628; doi:10.1038/s41467-022-28389-3)
Supplement: Supplementary file 3 — Reporting Summary [file 41467_2022_28389_MOESM3_ESM.pdf]

nature portfolio

Corresponding author(s): Anniina Färkkilä

Last updated by author(s): 12.01.2022

Reporting Summary

Nature Portfolio wishes to improve the reproducibility of the work that we publish. This form provides structure for consistency and transparency in reporting. For further information on Nature Portfolio policies, see our Editorial Policies and the Editorial Policy Checklist.

Statistics

For all statistical analyses, confirm that the following items are present in the figure legend, table legend, main text, or Methods section.

n/a	Confirmed	
☐	☒	The exact sample size (*n*) for each experimental group/condition, given as a discrete number and unit of measurement
☐	☒	A statement on whether measurements were taken from distinct samples or whether the same sample was measured repeatedly
☐	☒	The statistical test(s) used AND whether they are one- or two-sided<br>*Only common tests should be described solely by name; describe more complex techniques in the Methods section.*
☐	☒	A description of all covariates tested
☐	☒	A description of any assumptions or corrections, such as tests of normality and adjustment for multiple comparisons
☐	☒	A full description of the statistical parameters including central tendency (e.g. means) or other basic estimates (e.g. regression coefficient) AND variation (e.g. standard deviation) or associated estimates of uncertainty (e.g. confidence intervals)
☐	☒	For null hypothesis testing, the test statistic (e.g. F, t, r) with confidence intervals, effect sizes, degrees of freedom and P value noted<br>*Give P values as exact values whenever suitable.*
☒	☐	For Bayesian analysis, information on the choice of priors and Markov chain Monte Carlo settings
☒	☐	For hierarchical and complex designs, identification of the appropriate level for tests and full reporting of outcomes
☐	☒	Estimates of effect sizes (e.g. Cohen's *d*, Pearson's *r*), indicating how they were calculated

Our web collection on statistics for biologists contains articles on many of the points above.

Software and code

Policy information about availability of computer code

Data collection	ASHLAR (https://github.com/labsyspharm/ashlar)
Data analysis	Matlab version 2018b, R version 3.6.1, HistoCat v1.73, Fiji version 1.51, Ilastik version 1.3.2. Cycif Analysis Suite (https://github.com/yunguan-wang/cycif_analysis_suite). All custom code has been deposited in Github. https://github.com/farkkilab/pubs/tree/master/Launonen_et_al_2021.

For manuscripts utilizing custom algorithms or software that are central to the research but not yet described in published literature, software must be made available to editors and reviewers. We strongly encourage code deposition in a community repository (e.g. GitHub). See the Nature Portfolio guidelines for submitting code & software for further information.

Data

Policy information about availability of data

All manuscripts must include a data availability statement. This statement should provide the following information, where applicable:

- Accession codes, unique identifiers, or web links for publicly available datasets
- A description of any restrictions on data availability
- For clinical datasets or third party data, please ensure that the statement adheres to our policy

The datasets generated and analyzed during the current study are publicly available at Synapse ID syn23747228 [https://doi.org/10.7303/syn23747228]. Source data are provided with this paper. OncoPanel information is under restricted access and may be requested from the authors of Strickland et al. PMID: 26871470.

Field-specific reporting

Please select the one below that is the best fit for your research. If you are not sure, read the appropriate sections before making your selection.

☒ Life sciences ☐ Behavioural & social sciences ☐ Ecological, evolutionary & environmental sciences

For a reference copy of the document with all sections, see nature.com/documents/nr-reporting-summary-flat.pdf

Life sciences study design

All studies must disclose on these points even when the disclosure is negative.

Sample size	No sample size calculations were performed, as the samples had been already preselected.
Data exclusions	Pre-established quality control filters were used for the single cell data as described in the manuscript methods to remove cells with marker blobs, cells that had been lost or moved in between the t-CycIF cycles and cells that had a high expression of the background signal.
Replication	We had 1-3 TMA cores per patient as intrapatient replicates, and in total 31 BRCA1/2mut tumors as replicates for BRCA1/2mut tumors as well as 13 HRwt tumots as replicates for HRwt tumors.
Randomization	This was not a randomized study.
Blinding	Analyses could not be performed blindly.

Reporting for specific materials, systems and methods

We require information from authors about some types of materials, experimental systems and methods used in many studies. Here, indicate whether each material, system or method listed is relevant to your study. If you are not sure if a list item applies to your research, read the appropriate section before selecting a response.

Materials & experimental systems

n/a	Involved in the study	
☐	☒	Antibodies
☒	☐	Eukaryotic cell lines
☒	☐	Palaeontology and archaeology
☒	☐	Animals and other organisms
☒	☐	Human research participants
☒	☐	Clinical data
☒	☐	Dual use research of concern

Methods

n/a	Involved in the study	
☒	☐	ChIP-seq
☒	☐	Flow cytometry
☒	☐	MRI-based neuroimaging

Antibodies

Antibodies used	Antibodies with supplier name, catalog number and clone name as applicable are described in the manuscript in Supplementary Table 1.
Validation	https://www.cycif.org/antibodies/recommended is an online database with all relevant information on antibody validation on the antibodies used in our study.
